# Sex Hormone-Binding Globulin in Children and Adolescents

**DOI:** 10.4274/jcrpe.2764

**Published:** 2016-03-01

**Authors:** Banu Aydın, Stephen J. Winters

**Affiliations:** 1 University of Louisville Faculty of Medicine, Division of Endocrinology, Metabolism and Diabetes, Kentucky, USA

**Keywords:** Sex hormone-binding globulin, obesity, type 2 diabetes, metabolic syndrome, Non-alcoholic fatty liver disease, Polycystic ovary syndrome

## Abstract

Sex hormone-binding globulin (SHBG) is a circulating glycoprotein that transports testosterone and other steroids in the blood. Interest in SHBG has escalated in recent years because of its inverse association with obesity and insulin resistance, and because many studies have linked lower circulating levels of SHBG to metabolic syndrome, type 2 diabetes, nonalcoholic fatty liver disease, polycystic ovary syndrome, and early puberty. The purpose of this review is to summarize molecular, clinical, endocrine, and epidemiological findings to illustrate how measurement of plasma SHBG may be useful in clinical medicine in children.

**WHAT IS ALREADY KNOWN ON THIS TOPIC?**

Sex hormone-binding globulin (SHBG) is a glycoprotein produced in the liver that transports certain sex steroids in the circulation and regulates their access to target cells. Many studies have linked lower circulating levels of SHBG to obesity, type 2 diabetes, metabolic syndrome, non-alcoholic fatty liver disease, polycystic ovary syndrome, and early puberty.

**WHAT THIS STUDY ADDS?**

Our review was written to summarize the molecular, clinical, endocrine, and epidemiological findings which illustrate how measurement of plasma SHBG levels may be useful in clinical medicine in children. We believe that this review is novel and will be useful for the physicians who manage pediatric obesity and related comorbidities and for scientists who conduct translational research in this area.

## INTRODUCTION

Sex hormone-binding globulin (SHBG) is a 90-100 KDa homodimeric glycoprotein that is encoded by a single gene on the short arm of chromosome 17. Variable glycosylation explains the variation in molecular weight and is known to be increased by estrogens, but its significance is unknown. Circulating SHBG is produced primarily by hepatocytes, however, the gene is also expressed in the brain, uterus, prostate, breast, ovary, and testis (1), as well as in certain ovarian and prostate cancers. SHBG is found in the circulation of numerous mammals but is seemingly absent in the plasma of adult rats and mice, guinea pigs, and pigs. SHBG transports testosterone and other steroids in the blood plasma, reduces their metabolic clearance rate, and regulates their access to target tissues (2). While SHBG can sequester steroids from target tissues, there is some evidence that ligand-bound SHBG binds to membrane receptors, and stimulates cyclic adenosine monophosphate production (3), and/or enters cells by binding to the membrane protein megalin (4) to initiate a biological effect. Human SHBG binds dihydrotestosterone (DHT) > testosterone > estradiol as well as drugs such as levonorgestrel and fluoxymesterone (2).

Homozygous missense mutation resulting in a complete deficiency of plasma SHBG has been reported in a few cases. An affected adult male complained of low libido, decreased spontaneous morning erections, fatigue, muscular weakness, decreased shaving frequency, and had small testes and a low bone mass. His semen analysis was normal, however. His affected sister had delayed menarche, small breasts, and irregular menstrual periods (3). An adult woman with an undetectable level of SHBG and a compound heterozygote polymorphism had mild hirsutism that increased dramatically during a pregnancy when her free testosterone level was 4-fold elevated (4) suggesting that SHBG functions to protect the pregnant woman from placental hyperandrogenism. Polymorphisms have been reported that more subtly affect SHBG binding of testosterone.

**Figure 1:**
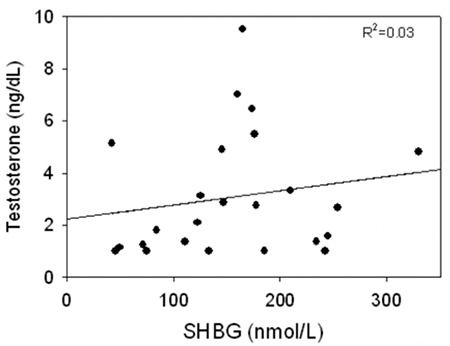
Relationship between sex hormone-binding globulin and total testosterone levels in prepubertal boys age 5-8 years, data from reference (82). SHBG: sex hormone-binding globulin

SHBG binds testosterone with high affinity (~1 nmol/L) and much of the SHBG-binding sites in adult male serum are occupied by testosterone such that the level of SHBG is a major determinant of the total testosterone level in adult men. Eugonadal adult men with low SHBG levels have low total testosterone levels, while men with high SHBG levels have higher testosterone levels. Obesity and hyperthyroidism, respectively, are examples of these effects. SHBG and testosterone are also related in newborn boys (5) during minipuberty but not in prepubertal boys with much lower testosterone levels in whom only a small portion of the SHBG in the plasma is occupied by testosterone (Figure 1).

## SEX HORMONE-BINDING GLOBULIN IN CHILDREN AND ADOLESCENTS

SHBG is present in the fetal circulation and in cord blood where levels are similar in males and females (6). SHBG levels are markedly increased in the maternal circulation due to the effect of placental estrogens, whereas the levels in cord blood are low and similar to values on day 2 of life. Whether SHBG plays a physiological role during fetal life is unknown. In one study of women from China, cord blood SHBG levels were lower among babies born to overweight mothers, most of whom had gestational diabetes (7).

**Figure 2:**
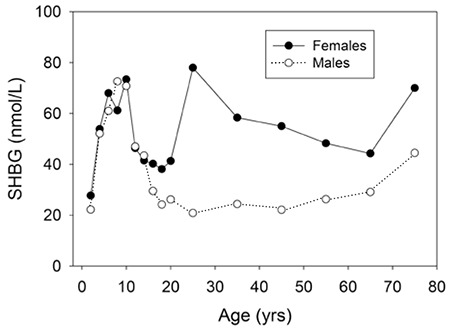
Sex hormone-binding globulin levels from birth to old age in males and females. Redrawn from Elmlinger et al (8). SHBG: sex hormone-binding globulin

As diagrammed in Figure 2, some cross-sectional studies indicate that SHBG levels rise substantially from birth to early childhood (8), whereas other studies indicate unchanged values (9). Longitudinal studies are lacking. During childhood, values are relatively stable but then decline at puberty, more so in boys than in girls (10). The reason for this change is not certain, but it is probably partly from androgens which are known to suppress SHBG levels (11). However, the decline is also seen in boys with idiopathic hypopituitarism (12) suggesting metabolic rather than neuroendocrine control. SHBG levels in adulthood are higher in women than in men, which is probably due to estradiol since estrogen administration is known to increase SHBG (13). Levels then rise slightly in the elderly, especially in men.

## REGULATION OF SEX HORMONE-BINDING GLOBULIN PRODUCTION

There is a 20-fold variation in SHBG levels among individuals, while the level of SHBG in a given individual is relatively constant (5). SHBG levels are unrelated to meals or time of day (6). Table 1 lists those factors that are known to influence the level of SHBG in blood. In most cases, the mechanism is unknown.

SHBG levels decrease with increasing obesity (14) and rise with weight loss (15). SHBG is reduced in type 2 diabetes mellitus (T2DM), and the strength of the association is reduced, but not eliminated, after adjustment for age and body mass index (BMI) (16). Notably, a low level of SHBG is a biomarker for the future development of the metabolic syndrome (MetS) (17), gestational diabetes (18), and T2DM (19).

Birkeland et al (20) reported that the level of SHBG represents an index of insulin resistance (IR), and many studies have confirmed this result (21). The traditional explanation for low SHBG levels in IR has been hyperinsulinemia (22). Studies have found an inverse correlation between SHBG and fasting (23), glucose-stimulated (24) or 24-hour mean insulin or C-peptide (25,26), and SHBG levels increase when IR improves and insulin levels decline with weight loss (27), resistance exercise (28), or following treatment with insulin sensitizing drugs (29). Moreover, adding insulin to HepG2 hepatocarcinoma cells reduced their production of SHBG (30,31), and insulin was reported to suppress SHBG messenger ribonucleic acid (mRNA) levels (31). A more recent study also using HepG2 cells, however, found no effect of insulin on SHBG secretion or mRNA levels. Instead, SHBG gene expression was reduced by the cytokines tumor necrosis factor-alpha (TNFα) (32) or interleukin-1 beta (IL1β) (33) and in transgenic mice that express SHBG after they were mated with obese, diabetic, hyperlipidemic db/db mice with inactivating leptin receptor mutation (34).

The nuclear receptor hepatic nuclear factor-4α (HNF4α)activates the promoters of many genes that are expressed in the liver and plays a key role in lipid metabolism (35). Functional HNF4α-binding sites are found in over 140 genes, including those involved in the metabolism of glucose, lipids, and amino acids, and in the proximal promoter of the SHBG gene. Moreover, over-expression of HNF4α in HepG2 cells by transient transfection increased the transcriptional rate of a SHBG-luciferase reporter (36). The effect of TNFα to suppress SHBG expression in vitro is mediated by HNF4α (37) and there is a strong correlation between the expression levels of HNF4α and SHBG in human liver (38). Thus, HNF4α regulation plays a central role in determining the level of SHBG in plasma.

**Figure 3:**
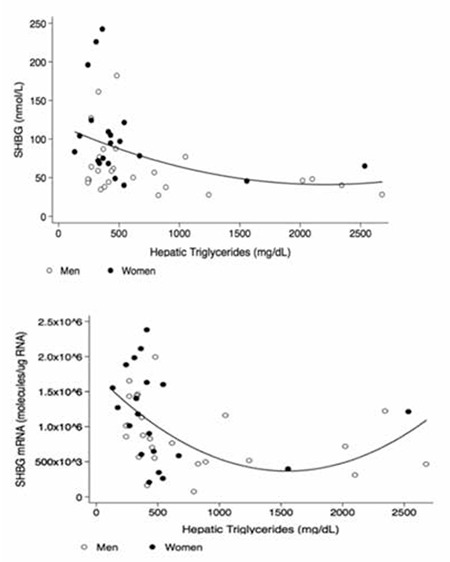
Associations between serum sex hormone-binding globulin levels (A) and sex hormone-binding globulin messenger ribonucleic acid (B) with hepatic triglyceride levels in women and men undergoing partial hepatectomy. Redrawn from reference (38). SHBG: sex hormone-binding globulin, mRNA: messenger ribonucleic acid

Hepatic fat is associated with IR (39) and recent studies have linked hepatic steatosis to low SHBG. A study of subjects at risk for T2DM which found no relationship between SHBG and insulin secretion following glucose challenge concluded that the amount of liver fat was the strongest predictor of SHBG (40). Several studies have subsequently found a strong inverse correlation between the amount of liver fat and serum levels of SHBG (41,42), and SHBG levels rise and liver fat decreases with weight loss (43). We recently found that serum SHBG and SHBG mRNA levels are low when the hepatic triglyceride concentration is elevated in a study of adult men and women undergoing hepatic resection as treatment for cancer (Figure 3) (38). In a recent study, Tong et al (44) reported that SHBG levels rose during short-term intensive insulin therapy in adults with newly-diagnosed T2DM which improved their lipid profiles and decreased liver enzymes [alanine aminotransferase (ALT), aspartate aminotransferase (AST), gamma glutamyltransferase (GGT)] and homeostatic model assessment-IR (HOMA-IR). Thus, the evidence to date suggests that excess hepatic fat is a key determinant of low SHBG, although more research is needed.

## CLINICAL DISORDERS AFFECTING SEX HORMONE-BINDING GLOBULIN

There are several disorders which affect SHBG levels, and understanding these effects may be important clinically.

## HYPER- AND HYPOTHYROIDISM

SHBG levels increase dramatically in hyperthyroidism in proportion to the levels of thyroxine (T4) and triiodothyronine (T3) in children (45) as well as in adults. Values normalize when hyperthyroxinemia is treated. High SHBG levels will result in elevated levels of testosterone in both males and females, and may present a diagnostic challenge and lead to an unneeded evaluation for pituitary, adrenal, or gonadal disorders. High SHBG leads to elevation of luteinizing hormone (LH) and estradiol and may produce breast enlargement in males (46). High SHBG levels result from thyroid hormone activation of the HNF4α gene promoter which, in turn, stimulates SHBG expression (47). SHBG is thus a marker of increased thyroid hormone bioactivity. This idea has been used clinically in patients with inappropriate thyroid-stimulating hormone (TSH) secretion with high free T4/T3 levels and TSH levels that are not suppressed. Some of these individuals have inactivating mutations of the thyroid hormone receptor, which disrupts feedback control of TSH secretion. These individuals may be recognized by their normal SHBG levels (48) and distinguished from patients with TSH-producing pituitary tumors who have hyperthyroxinemia and high SHBG (49). SHBG levels are reduced in hypothyroidism, which in men may be interpreted as testosterone deficiency.

## ADRENAL DISORDERS

SHBG levels are reduced in patients with Cushing’s syndrome (50) and in patients treated with glucocorticoids (51). Low SHBG together with adrenocorticotropic hormone (ACTH)-mediated testosterone production may cause virilization in children, and contribute to delayed puberty, and anovulation and oligo-amenorrhea in ACTH-dependent Cushing’s syndrome (52). In children treated with prednisone or dexamethasone for leukemia, the fall in SHBG occurred slowly over 4 weeks during which time BMI and leptin levels rose suggesting a connection to IR (51). Perhaps because of a tendency to abdominal adiposity (53), SHBG levels are also low in girls with congenital adrenal hyperplasia (54).

## PITUITARY DISORDERS

SHBG levels are elevated in patients with growth hormone deficiency (55) and are decreased in patients with acromegaly. To what extent these changes are mediated by insulin sensitivity and resistance is unknown. Lower SHBG levels have been reported in patients with hyperprolactinemia, but this association may also be influenced by the higher body fat with hypogonadism.

## LIVER DISEASE

SHBG is produced in the liver, and SHBG levels are affected by diseases of the liver through a variety of mechanisms. SHBG levels are elevated in patients with alcoholic cirrhosis. Alcohol damages the testis so that LH levels are elevated which in turn stimulate testicular aromatase and thereby estradiol production which increases SHBG. High SHBG may also be due to increased estrone and estradiol from the adrenals that is activated by stress and ACTH (56). Moreover, sulfatase (the enzyme which converts inactive estrogen sulfates to active estrogens) is increased in alcoholic liver. Amenorrheic women with both alcoholic and nonalcoholic cirrhosis, by contrast, tend to have low LH/follicle stimulating hormone and normal SHBG levels (57). SHBG levels are also markedly increased with hepatitis-B or hepatitis-C infection (58), while patients with liver disease due to hemochromatosis develop hypogonadotropic hypogonadism due to pituitary iron deposition and tend to have slightly elevated SHBG levels. Non-alcoholic fatty liver disease (NAFLD), a condition of increased hepatic triglycerides in the absence of excess alcohol consumption, is associated with increased visceral adipose tissue (VAT), IR, and dyslipidemia, and with low SHBG levels (41).

## OBESITY AND RELATED COMORBIDITIES

**Figure 4:**
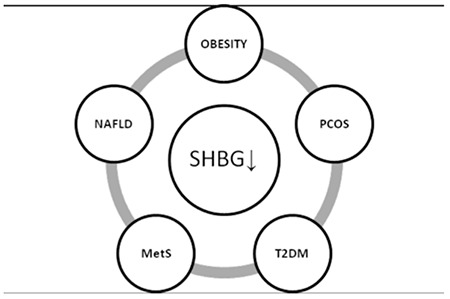
Relationship between obesity-related comorbidities and sex hormone-binding globulin levels. MetS: metabolic syndrome, NAFLD: non-alcoholic fatty liver disease, PCOS: polycystic ovary syndrome, T2DM: type 2 diabetes mellitus, SHBG: sex hormone-binding globulin

Childhood obesity is one of the most important health problems of our era due to its high prevalence and association with many chronic diseases and shorter life expectancy (59,60). Recent studies have found alarming increases in the rates of childhood obesity and related comorbidities, such as T2DM, MetS, peripubertal hyperandrogenemia (HA), polycystic ovary syndrome (PCOS), NAFLD, and early puberty (59,61,62,63,64). These disorders are inter-related, and their etiology and pathogenesis are multifactorial and controlled by genetic factors, the intrauterine environment, and an unhealthy lifestyle (63). Since these conditions increase the risk of early cardiovascular disease (CVD), finding effective ways to identify at-risk children as early as possible is an important goal. SHBG is a promising biomarker (Figure 4) because SHBG levels are unaffected by meals or time of day, there is no influence of sex hormones in prepubertal children, and SHBG can be readily measured in a finger-stick blood sample. Many studies have linked lower circulating levels of SHBG to obesity, IR, MetS, T2DM, PCOS, and NAFLD (21,60,65). These associations may be explained by the idea that low SHBG is a marker for IR.

## TYPE 2 DIABETES MELLITUS AND SEX HORMONE-BINDING GLOBULIN

SHBG levels are low in adults with T2DM, and many studies show that low levels predict diabetes risk (19,66,67). The relationship is reduced, but maintained, after controlling for obesity. T2DM is increasingly diagnosed in children as young as age 10, and now accounts for 20% to 50% of new-onset diabetes in children (64). In the U.S., it disproportionately affects Latino and Black children. Several studies showed that weight loss through calorie restriction and metformin treatment, in combination with lifestyle changes, increases serum SHBG levels in adolescents at risk for developing diabetes (68). In those studies, insulin levels decreased with intervention due to improvement in insulin sensitivity.

It has been suggested that SHBG may have a causal role in the risk of T2DM since Mendelian randomization studies have reported that carrying specific SHBG single-nucleotide polymorphisms (SNPs) affects the risk of T2DM (19,66). Carriers of rs6259 polymorphism were shown to have higher SHBG levels and a lower risk of T2DM, and rs6257 SNP carriers were reported to have lower SHBG levels and higher risk of T2DM (19). In another larger study including 86138 adults, presence of the rs1799941 SNP was associated with increased SHBG concentrations and reduced risk of T2DM after correction for age, sex, and BMI (66). In a recent study, Wang et al (69) showed that circulating SHBG levels were predictive for future IR in healthy young Finnish adults, whereas Mendelian randomization suggested minor, if any, causal effects.

## METABOLIC SYNDROME AND SEX HORMONE-BINDING GLOBULIN

MetS is a combination of risk factors for increased CVD morbidity and mortality that includes central obesity, hypertension, dyslipidemia, and impaired glucose metabolism. MetS is increasingly recognized in children and adolescents, but the diagnostic criteria for this age group remain controversial (70). Furthermore, no accepted definition applies to all ethnic groups because ethnic variations exist in the distribution of MetS components in children (71,72,73,74).

As in adults, SHBG levels are low in children and adolescents diagnosed with MetS (75,76,77). In a cross-sectional study of 815 school children in Spain by de Oya et al (77), SHBG levels were lower in those adolescents with MetS or with some MetS features, such as abdominal obesity, high blood pressure or high insulin and low high density lipoprotein cholesterol (HDL-C) levels. Agirbasli et al (78) reported that low SHBG was a significant predictor of low HDL-C levels in Turkish children and adolescents. Detailed metabolic profiling of 6475 young adults from two population-based Finnish cohorts revealed a strong association between SHBG and circulating lipids and metabolites reflecting the degree of adiposity and IR. Low SHBG predicted the development of IR in early adulthood, and these associations remained robust after adjustment for baseline adiposity, insulin and testosterone levels (69). Glueck et al (65) demonstrated that low SHBG levels in U.S. schoolgirls at age 14 were a positive predictor for the development of MetS 10 years later. Thus, SHBG may be valuable biomarker for MetS risk in children long before the disease progresses.

**Figure 5:**
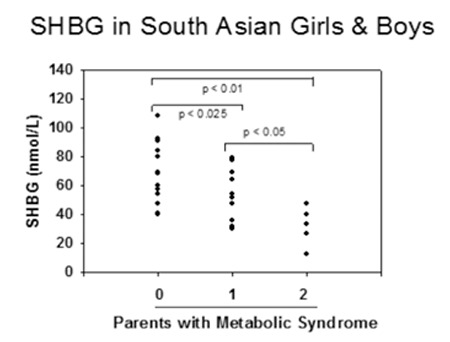
Serum levels of sex hormone-binding globulin in South Asian Indian children according to the diagnosis of Metabolic syndrome in their parents; redrawn from reference (86). SHBG: sex hormone-binding globulin

There are substantial racial and ethnic differences in body composition for a given BMI between subjects of the same sex and age. Previous studies have documented a genetic predisposition for MetS (79,80) and there is evidence that SHBG levels, like MetS components, vary by ethnicity (Table 2). In the national health and nutrition examination survey (NHANES) study, SHBG levels were lower in Mexican-American males age 12-19 than in non-Hispanic blacks and whites (81). Abdelrahaman et al (82) found that high levels of SHBG are more common among healthy African American prepubertal boys, a racial group with more subcutaneous but less VAT than their white peers. Hergenc et al (83) reported that Turkish middle-aged adults have lower SHBG levels compared with Germans, and most of the difference in HDL-C between Germans and Turks was explained by ethnicity independent of obesity markers, insulin, and sex hormones. The MELEN study of 751 Turkish adult women and men, with a mean age of 55 years, found that 34% had MetS (84), while a recent study of German women reported a prevalence of 23.1% (85). South Asian Indians are an ethnic group at especially high risk for MetS and T2DM even though they have low BMI. Krishnasamy et al (86) found that prepubertal South Asian Indian children with one parent with MetS had 24% lower SHBG levels, and with both parents affected had 55% lower SHBG levels (Figure 5). Their study also demonstrated that SHBG levels were inversely related to waist circumference and to BMI percentile in those children. Significant associations were reported between SHBG (rs6257), cholesterol ester transfer protein (rs708272) polymorphisms and high triglycerides, low HDL-C and high low-density lipoprotein cholesterol levels in a cohort of 365 Turkish children and adolescents (87). Additionally, White et al (88) reported that SNPs located in the SHBG gene (rs1799941) were associated with MetS in children. They found that association with MetS remained after sequential adjustment for each MetS component, indicating that the identified association was not being driven by any single trait. The A allele of rs1799941 was associated with a significant increase in SHBG levels in control subjects, while there was no association between rs1799941 and SHBG levels in children with MetS.

## PERIPUBERTAL HYPERANDROGENAEMIA, ADOLESCENT POLYCYSTIC OVARY SYNDROME AND SEX HORMONE-BINDING GLOBULIN

PCOS is the most common endocrine disorder among reproductive-aged women, and the most common cause of infertility in young women. PCOS is characterized by HA, menstrual dysfunction, and polycystic ovarian morphology, and arises as a complex trait due to inherited and environmental factors. Adolescents with PCOS are more insulin resistant and hyperinsulinemic compared to weight-matched non-hyperandrogenemic girls (89). SHBG levels are reduced in PCOS resulting in a higher portion of biologically active androgen, and an increased number of (TAAAA)n repeats in the SHBG promoter region may be a susceptibility locus for PCOS (90) although this association is controversial (91). The clinical manifestations of PCOS often begin during puberty, but the anovulation and acne that often occur in healthy teenage girls make the PCOS diagnosis challenging in adolescents. Therefore, biochemical evidence of HA, including low SHBG levels, is important in the evaluation of adolescent PCOS (92).

SHBG levels are low in obese and overweight peripubertal girls, and weight loss is associated with a decrease in testosterone and an increase in SHBG levels (93,94). Not all peripubertal obese girls have elevated androgens, however, and not all adolescents diagnosed with PCOS are obese or overweight, suggesting that obesity per se is not sufficient to produce HA (95). Teenage daughters of PCOS patients are more likely to have features of the MetS, and to be hyperinsulinemic (96), have larger ovaries beginning at Tanner stage 1, and by Tanner stage V have lower SHBG levels than daughters born to control women (97).

The prevalence of PCOS in women born small for gestational age (SGA) is twice as high as in women born with normal weight (98). Girls born SGA with catch-up growth were shown to display more visceral fat as compared to age- and BMI- matched children born at normal weight (99) and have lower SHBG concentrations and an exaggerated adrenarche between the ages of 6 and 8 years (100). Longitudinal studies revealed that metformin-treated low birth weight children were leaner, had less IR and higher SHBG levels than placebo-treated children, and in low birth-weight girls, the increase in SHBG was followed by a delay of menarche (68).

## NON-ALCOHOLIC FATTY LIVER DISEASE AND SEX HORMONE-BINDING GLOBULIN

NAFLD has become the most common form of liver disease in childhood. The presence and severity of NAFLD are associated with an increased incidence of CVD, independent of established risk factors, and NAFLD was suggested as not only a marker of CVD risk but also an important player in CVD pathogenesis (101). Early diagnosis and treatment is crucial, but most children with NAFLD remain undiagnosed (63). While liver biopsy is the gold standard for diagnosis, the European Society for Pediatric Gastroenterology, Hepatology and Nutrition instead recommends abdominal ultrasound and liver function tests for all obese children (102). Large-scale ultrasound may not be a cost-effective approach, however, and liver transaminase (ALT, AST) elevations were a poor predictor, especially in the earliest stages (60,102). Additionally, the serum GGT level may be a marker of oxidative stress rather than a specific marker of NAFLD-induced liver disease (103).

Previous studies have shown that liver fat is a stronger predictor of SHBG than is total body fat. Serum SHBG levels were lower in high-grade NAFLD patients with T2DM than in diabetics without NAFLD (104), and lower SHBG levels were found in adult (105) and adolescent (106) PCOS subjects with NAFLD compared with PCOS subjects without NAFLD (40). Moreover, those women with PCOS and NAFLD are more insulin resistant than are PCOS women without evidence for hepatic steatosis by ultrasound (107). Wolfgram et al (108) showed significant correlations between hepatic proton density fat fraction measured by magnetic resonance imaging and SHBG blood levels in non-obese Hispanic middle school girls. Finally, SHBG levels were shown to rise as liver fat decreases with weight loss (43). In the light of these findings, SHBG represents an alternative marker for pediatric NAFLD risk stratification and in certain children at higher risk for NAFLD and MetS, may be a useful biomarker perhaps prior to the development of obesity.

## EARLY PUBERTY AND SEX HORMONE-BINDING GLOBULIN

There is accumulating evidence that puberty in girls is occurring at an earlier age, and the obesity epidemic is an important factor in this phenomenon (109). SHBG may function during childhood to restrict the actions of sex steroids until puberty at which time sex steroid levels increase in concert with a fall in plasma SHBG levels such that the overall result is a progressive increase in both total and free and hormone levels. The mechanism for the decline in SHBG at puberty is not well understood but appears to be metabolic rather than hormonal since the decrease occurs in boys with hypopituitarism (11). Moreover, insulin sensitivity declines in early normal puberty (110) which could lead to lower SHBG levels. In a cross-sectional study on 132 healthy Caucasian children and adolescents, SHBG was a strong predictor of insulin sensitivity after adjustment for puberty, fat mass, and aerobic fitness (111). In that study, the authors reported a significant negative association between metabolic risk and SHBG levels after adjustment for relevant confounders, and hypothesized that SHBG integrates the marked changes in glucose metabolism and body composition that occur during the pubertal transition and might be valuable in the assessment of CVD risk during puberty. Pinkney et al (10) reported that girls with lower SHBG levels at 5 years of age reached Tanner stage 2 earlier, tended to have earlier increases in LH secretion, and an earlier age at peak height velocity and menarche. They reported negative correlations between SHBG and adiposity, insulin, IGF-I, CRP, and leptin, and positive associations between adiponectin and SHBG (10). Sorensen et al (112) found that, after adjustment for BMI and pubertal stage, girls with central precocious puberty have lower SHBG levels compared with healthy controls, and the decline in SHBG levels during puberty is associated with increasing fat mass in healthy children and adolescents.

Although studies tend to indicate a relationship between obesity and early puberty in girls, the association in boys is controversial. Some authors report advanced sexual maturation in obese boys (113,114), some describe normal pubertal timing (115), while others report delayed testicular development with obesity (116,117). The reason for these contradictory findings is uncertain but might be due to differences in the study populations, pubertal markers, and cut-off points for defining obesity. Studies have shown that SHBG levels are lower in obese boys than in their normal weight peers (23,115). Pinkney et al (10) reported that boys with lower SHBG levels at age 5 years reached Tanner stage 2 earlier, but there was no relationship between SHBG and earlier onset of LH secretion or age at peak height velocity. Most studies report lower total testosterone levels in obese boys during pubertal progression (118) which can be explained by lower SHBG whereas SHBG and total testosterone are unrelated in prepubertal boys (82).

Denburg et al (119) reported lower SHBG levels and decreased insulin sensitivity in boys with premature pubarche (PP) than in age- and BMI-matched peers. They showed significant correlations between SHBG and measures of insulin sensitivity in boys with PP and controls, and suggested that SHBG may be a marker for IR. On the other hand, Potau et al (120) found that SHBG levels and measures of the glucose and insulin response to an oral glucose challenge were comparable in boys with PP and controls, and concluded that PP in boys may be regarded as a variant of normal development.

In conclusion, evidence is accumulating that low SHBG levels are an indicator of IR, and SHBG may be an easy-to-measure and clinically useful biomarker for the early identification of children who are destined to develop obesity-related chronic diseases. Further research is needed to understand how SHBG is regulated in children. Moreover, studies with respect to race and ethnicity are needed to establish SHBG reference ranges for children and adolescents. Finally, whether SHBG is solely a biomarker or rather participates actively in the pathogenesis of metabolic disease remains to be elucidated.

Peer-review: Internal peer-reviewed.

## AUTHORSHIP CONTRIBUTIONS

Concept: Stephen J. Winters, Design: Stephen J. Winters, Data Collection and/or Processing: Stephen J. Winters, Banu Aydın, Analysis and/or Interpretation: Stephen J. Winters, Banu Aydın, Literature Research: Stephen J. Winters, Banu Aydın, Writing: Stephen J. Winters, Banu Aydın.

Financial Disclosure: BA is supported through The Scientific and Technical Research Council of Turkey (TUBITAK) (International Postdoctoral Research Scholarship Program). SJW is supported in part by a gift from the Walter F. and Avis Jacobs Foundation.

## Figures and Tables

**Table 1:**
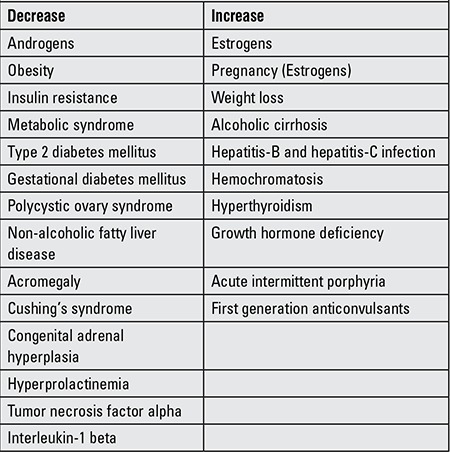
Factors that influence the level of sex hormone-binding globulin in blood

**Table 2:**
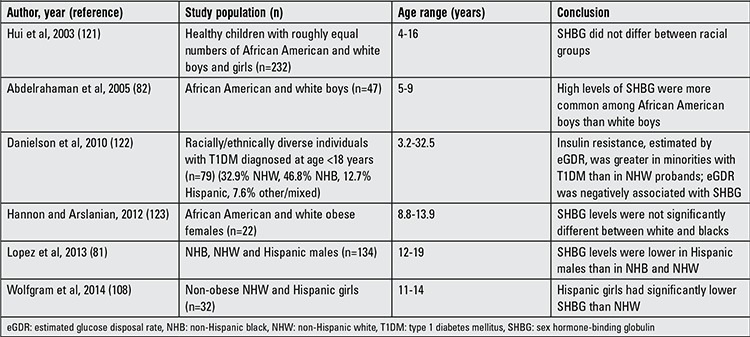
Studies evaluating ethnic and racial differences in serum sex hormone-binding globulin levels in children and adolescents
